# Harnessing large language models for reliable automatic Charlson Comorbidity Index scoring: a validation study

**DOI:** 10.1093/jamiaopen/ooag212

**Published:** 2026-11-02

**Authors:** Lana Mei Borcard, Anna-Katharina Calek, Michael Rebsamen, Benedikt Herzog, Sebastiano Caprara, Mazda Farshad, David Ephraim Bauer

**Affiliations:** Department of Orthopaedics, Balgrist University Hospital, University of Zurich, 8008 Zurich, Switzerland; Department of Orthopaedics, Balgrist University Hospital, University of Zurich, 8008 Zurich, Switzerland; Department of Orthopaedics, Balgrist University Hospital, University of Zurich, 8008 Zurich, Switzerland; Department of Orthopaedics, Balgrist University Hospital, University of Zurich, 8008 Zurich, Switzerland; Department of Orthopaedics, Balgrist University Hospital, University of Zurich, 8008 Zurich, Switzerland; Department of Orthopaedics, Balgrist University Hospital, University of Zurich, 8008 Zurich, Switzerland; Department of Orthopaedics, Balgrist University Hospital, University of Zurich, 8008 Zurich, Switzerland

**Keywords:** large language models, Charlson Comorbidity Index, electronic health records, natural language processing, comorbidity extraction

## Abstract

**Objectives:**

To determine whether large language models can compute the Charlson Comorbidity Index (CCI) from unstructured, German-language diagnosis lists with reliability approaching that of expert human consensus.

**Materials and Methods:**

We retrospectively analyzed the complete free-text diagnosis lists of 200 patients from a tertiary orthopedic center, stratified by American Society of Anesthesiologists (ASA) physical status I-IV. Two board-certified orthopedic surgeons and 1 chiropractor independently assigned CCI values; a consensus reading served as the reference standard. Twelve open-source models, hosted on-premise, and 5 commercial models were each evaluated with and without guideline-based prompting. Agreement was quantified by accuracy, Cohen kappa, and the coefficient of determination, and discrepancies were classified using an adapted error taxonomy.

**Results:**

Human interrater reliability was excellent (intraclass correlation 0.97). Across models, accuracy against consensus ranged from 0.34 to 0.79 and kappa from 0.27 to 0.75. The best open-source model (GPT-OSS-120B with guidelines) matched the best commercial model (coefficient of determination 0.93). Agreement declined with increasing ASA class, falling to 0.34-0.56 in ASA IV. Most discrepancies were knowledge-related (41.7%-58.3%); computation errors were rare, and 22.2%-32.0% arose from diagnosis lists too incomplete for any classification.

**Discussion:**

Performance approached expert level in low-complexity records but degraded as documentation complexity increased, indicating that record quality rather than model reasoning is the dominant constraint. Locally hosted open-source models performed comparably to proprietary systems, allowing comorbidity extraction without transferring data off-premise.

**Conclusion:**

Large language models can approximate expert CCI scoring directly from unstructured German-language clinical text, supporting scalable risk adjustment where documentation is adequate.

## Introduction

Accurately capturing patient comorbidities is central to clinical research, risk adjustment, and outcome prediction. The Charlson Comorbidity Index (CCI) remains one of the most widely used instruments for quantifying comorbidity burden and predicting mortality across diverse populations and care settings.^1^^,^^2^ Beyond mortality prediction, the CCI is increasingly applied for cohort selection and stratification in clinical and epidemiological studies.^1^^,^^2^ However, in real-world electronic health record (EHR) data, CCI scores are rarely available. Manual computation requires detailed review of patient diagnoses across 17 disease categories—an approach that, while clinically robust, is time-consuming and vulnerable to inconsistencies arising from differences in coding practices, ICD (International Classification of Diseases) versions, and subjective interpretation.^1^^,^^2^

Large language models (LLMs) offer a promising alternative for automating comorbidity extraction. Trained on vast corpora of text, these models can process unstructured medical narratives rapidly, standardize coding practices, and potentially reduce variability while improving reproducibility.^3^^,^^4^ Early studies suggest that LLMs can achieve performance comparable to human experts in extracting comorbidity information.^4^^,^^5^ Nevertheless, concerns remain regarding their domain adaptation, robustness, and potential biases, particularly in real-world, unstandardized diagnosis lists and in languages other than English, for which comparatively few clinical evaluations exist.^4–6^

Despite the broad use of the CCI and the rapid integration of LLMs into biomedical research, few studies have directly compared LLM-based CCI calculation with human expert ratings in real-world EHR data, and none, to our knowledge, in German-language records.^7^ This represents a critical gap, as real-world clinical documentation often includes narrative elements, abbreviations, and local conventions that can challenge both automated and manual approaches.^4^^,^^5^

This study addresses this gap by evaluating whether modern LLMs can compute the CCI from unstructured, German-language EHRs. We hypothesized that LLMs could approximate expert-level CCI scoring while substantially reducing manual effort. By benchmarking multiple commercial and open-source LLMs against human consensus ratings and systematically analyzing sources of disagreement, this study aims to determine the feasibility of scalable, reproducible comorbidity quantification from German-language clinical documentation—thereby advancing automated risk adjustment and outcome modeling.

## Materials and methods

In this retrospective study, we analyzed the medical records of 200 patients in a tertiary care center specialized in the treatment of orthopedic patients. Prior to the full-scale analysis, we conducted a trial run on a subset of 100 patients to familiarize the investigators with the CCI classification process and refine the prompting strategy for the LLMs. Using the insights gained from this preliminary run, we proceeded with the full extraction of diagnostic data for all 200 patients, randomly sampled from the EHR records. The diagnoses were then independently classified by 2 board-certified orthopedic surgeons and 1 chiropractor. Finally, a consensus reading was performed to reconcile disagreements and establish the final CCI classifications.

### Study design

This retrospective study evaluated the agreement between LLMs and human experts in calculating the CCI from unstructured German diagnosis lists.

### Patient selection, data sources, and data anonymization

A total of 200 patients were randomly selected from hospital admissions within the past 6 months, with stratification by the American Society of Anesthesiologists (ASA) physical status classification to ensure balanced representation across categories I-IV. For each case, the complete diagnosis list was retrieved from the electronic hospital database using an automated script. These diagnoses, documented in German by attending physicians during routine clinical practice, frequently consisted of narrative descriptions rather than standardized coding. To comply with data protection regulations, no patient identifiers or personally identifiable information were collected. Each case was instead assigned a unique study identifier, and only the diagnosis lists, and age category were extracted. Age was grouped into 5 predefined categories (<50 years, 50-59 years, 60-69 years, 70-79 years, ≥80 years). Before analysis, all data underwent an additional review to exclude any inadvertent inclusion of identifiable information.

### Human rater

The reference standard for CCI was established by 3 independent human raters: 2 board-certified orthopedic surgeons and 1 chiropractor. To calibrate interpretation, a pilot dataset of 100 cases was reviewed jointly before the main analysis. Discrepancies were discussed and reconciled, and classification rules were finalized. For the main dataset, raters independently assigned CCI values, followed by a consensus reading to generate the ground truth for subsequent comparisons.

### Large language models

A range of the most recent commercial and open-source LLMs were included (see full list in Appendix Table S1). Each model was tested in 2 configurations: (1) using the unstructured German diagnosis lists and the age group with its CCI points, without the CCI criteria, and (2) using the same lists accompanied by the CCI guidelines developed by the human raters during the trial run (reproduced in full in Box S1). The open-source models were hosted on-premise using the ollama framework (https://ollama.com) running on an NVIDIA DGX with A100 GPUs (NVIDIA Corporation). To maximize the reproducibility of the results, a temperature = 0 parameter was used where supported by the models. To estimate the variability of the LLM-derived results, a separate experiment was conducted by sampling 24 results with temperature = 0.1 and a different seed parameter for each sample.

### Statistical analysis

Statistical analyses were conducted using R (version 4.3.2) and Python (version 3.13). Interrater agreement was assessed by calculating the intraclass correlation coefficient (ICC, 2-way/agreement) among the 3 experts as well as the coefficient of determination (*R*^2^) between each pair of raters.

Model performance was evaluated against the human consensus using multiple agreement and reliability measures, including overall accuracy, coefficient of determination (*R*^2^), and Cohen’s *κ*. These metrics captured both classification accuracy and concordance with expert ratings.

All raters and all models reported the CCI as a single integer score (age-adjusted CCI, ie, including the age points; observed consensus scores ranged from 0 to 13); no grouping into categories was applied. Accuracy was defined as the proportion of cases in which the LLM score exactly matched the consensus score. Cohen’s *κ* was computed treating each integer CCI value as a nominal category (unweighted *κ*), and therefore represents a strict, chance-corrected measure of exact agreement. Because exact-match metrics do not distinguish a 1-point from a multipoint deviation, we additionally report the coefficient of determination (*R*^2^, computed as 1 − SS_res_/SS_tot_ (SS_res_ = the sum of squared differences between predicted and consensus scores, SS_tot_ = the sum of squared differences between consensus scores and their mean) with the consensus score as reference, so that negative values indicate predictions less accurate than the mean consensus score). The same pairwise metrics (accuracy, *κ* and *R*^2^) were computed between each pair of human raters (Table 2), so that human-human and LLM-human agreement are directly comparable; the ICC was reported additionally as a single summary of absolute agreement across all 3 raters.

**Table 1.** Baseline characteristics of the study population (*n* = 200).

Age group	ASA I	ASA II	ASA III	ASA IV	Total *n* (%)
**<50 years**	43	18	5	2	68 (34.0)
**50-59 years**	4	6	10	6	26 (13.0)
**60-69 years**	3	17	14	9	43 (21.5)
**70-79 years**	0	8	7	22	37 (18.5)
**≥80 years**	0	1	14	11	26 (13.0)

Abbreviation: ASA, American Society of Anesthesiologists.

**Table 2.** Interrater agreement.

	R1/R2	R1/R3	R2/R3
**Accuracy**	0.84	0.86	0.89
**Kappa**	0.81	0.83	0.87
***R* ^2^**	0.92	0.96	0.94

Accuracy, Cohen’s kappa, and *R*^2^ for agreement between the 3 expert raters on the test dataset.

To characterize the nature and origin of discrepancies between LLM-derived and human consensus CCI scores, we conducted a structured review following an adapted version of the error taxonomy proposed by Khandekar et al.^8^ For each discrepant case, the reasoning of both the LLM and the human consensus was reexamined to determine the correct CCI classification. This review was performed jointly by the 3 human raters (D.E.B., A-K.C., and L.M.B.), following the same procedure used to establish the human consensus: for each case, the raters determined whether the LLM or the human consensus had deviated from the CCI definitions, using the original CCI publication and its subsequent updates as reference, and agreed on the correct classification by consensus. The reviewers were not blinded to whether a score originated from the LLM or from the human consensus. This process aimed to objectively identify the mechanism of error rather than assign responsibility to either source.

Each case was then categorized into 1 of 4 error types according to the adapted taxonomy:

Type A (knowledge errors): incorrect or incomplete application of CCI rules;

Type B (extraction errors): inaccurate extraction or interpretation of relevant diagnostic information from the input list;

Type C (computation errors): correct identification of comorbidity parameters but incorrect arithmetic or weighting; and

Type D (other errors): all remaining misclassifications that could not be clearly assigned to types A-C.

## Results

### Patient characteristics

A total of 200 patients were included. Distribution across age groups was as follows: <50 years (*n* = 68, 34.0%), 50-59 years (*n* = 26, 13.0%), 60-69 years (*n* = 43, 21.5%), 70-79 years (*n* = 37, 18.5%), and ≥80 years (*n* = 26, 13.0%). Stratification by ASA physical status classification was balanced, with 50 patients (25.0%) in each of ASA I-IV. The cross-distribution of age and ASA categories is summarized in Table 1.

### Interrater reliability

Interrater agreement between the 3 human raters (R1-R3) was consistently high across all metrics (cf. Table 2). Accuracy ranged from 0.84 (R1 vs R2) to 0.89 (R2 vs R3). Cohen’s kappa values indicated substantial to almost perfect agreement, with coefficients of 0.81 (R1 vs R2), 0.83 (R1 vs R3), and 0.87 (R2 vs R3). Agreement on the CCI scores was similarly strong, with coefficients of determination (*R*^2^) of 0.92 (R1 vs R2), 0.96 (R1 vs R3), and 0.94 (R2 vs R3). The overall ICC for absolute agreement among all 3 raters was 0.97, confirming excellent interrater reliability.

When stratified by ASA score (Table 1), interrater agreement remained high across all categories. For ASA I, full concordance was observed between all rater pairs (*R*^2^ = 1.00, ICC = 1.00). Agreement slightly declined with increasing ASA class, showing *R*^2^ values of 0.97-0.98 (ICC = 0.99) for ASA II, 0.84-0.93 (ICC = 0.94) for ASA III, and 0.73-0.87 (ICC = 0.88) for ASA IV. Overall, despite minor reductions in higher ASA categories, reliability metrics remained within the range of substantial to almost perfect agreement.

### LLM-human consensus agreement

A total of 12 open-source and 5 commercial LLMs were evaluated with and without guideline-based prompting. Across models, agreement in calculated CCI scores was generally high, with most pairwise comparisons yielding *R*^2^ values above 0.85. Accuracy tended to improve when guideline-based instructions were provided (Figure 1).

**Figure 1.**
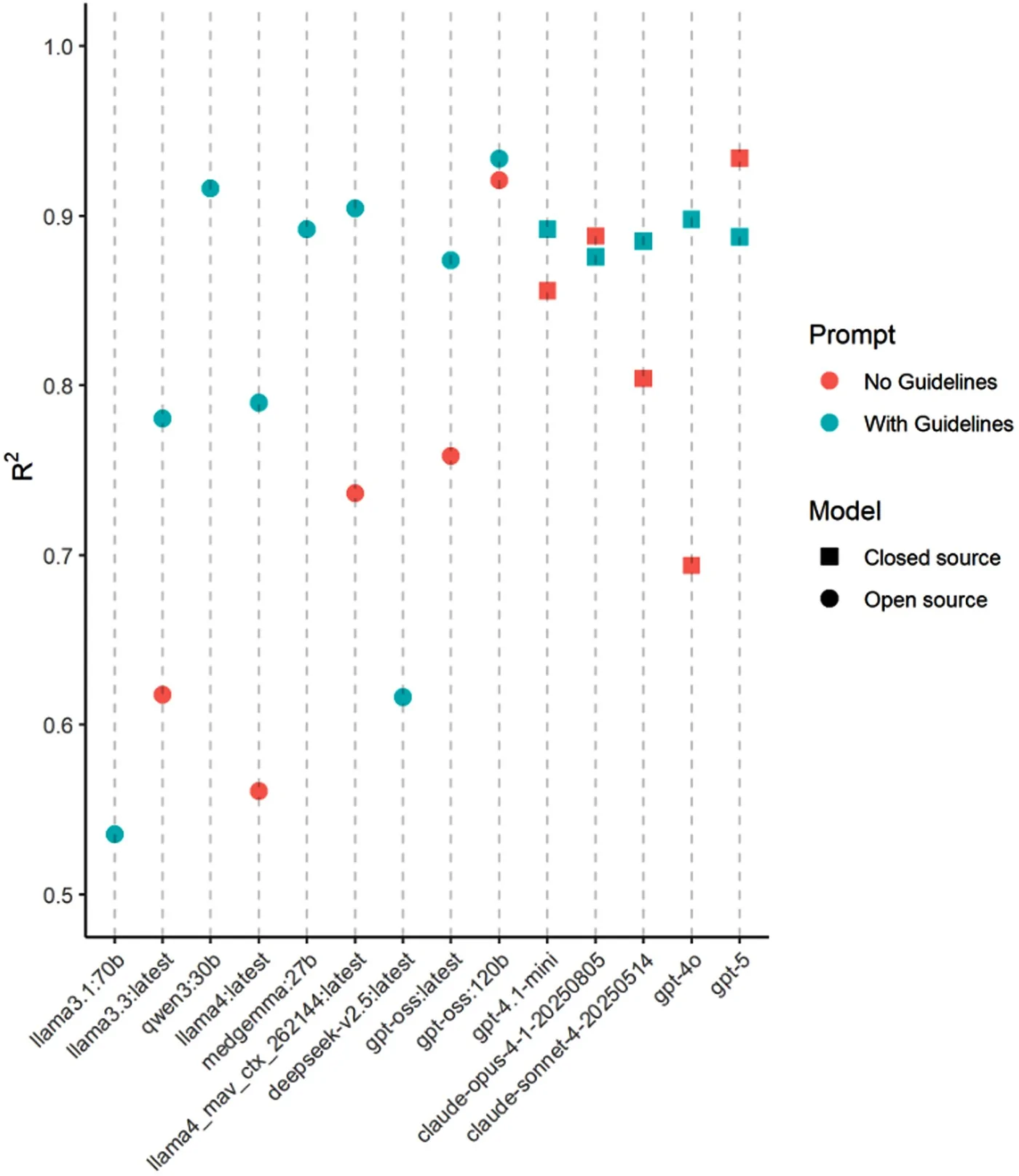
Overview of results. Performance of various models for the test dataset. *R*^2^ values represent the coefficient of determination between the estimated CCI (Charlson Comorbidity Index) generated by the LLMs (large language models) and the consensus of the human expert raters.

Closed-source models such as GPT-4 (OpenAI), Claude (Anthropic PBC), and GPT-5 (OpenAI) showed particularly strong concordance with human consensus (*R*^2^ ≈ 0.70-0.93), largely unaffected by prompting strategy (*R*^2^ ≈ 0.788-0.90). In contrast, open-source models displayed greater variability: smaller LLaMA variants achieved only moderate agreement without structured instructions (*R*^2^ ≈ 0.55-0.75) but improved substantially under guideline-based prompting. Larger open-source models like LLaMA 4 Maverick (Meta Platforms, Inc.) and GPT-OSS-120B (OpenAI), demonstrated high consistency with both open- and closed-source peers when guidelines were applied.

When compared against the human consensus, open-source models reached accuracy values ranging from 0.34 (DeepSeek-R1:70b; DeepSeek AI, without guidelines) to 0.79 (GPT-OSS-120B with guidelines). *R*^2^ values extended from –0.26 to 0.93, and Cohen’s *κ* ranged from 0.27 to 0.75. For commercial models, accuracy values spanned 0.49 (Claude-Sonnet-4; Anthropic PBC, without guidelines) to 0.76 (GPT-4.1-mini; OpenAI, with guidelines). *R*^2^ values were consistently high (0.80-0.93), and Cohen’s *κ* ranged between 0.41 and 0.73.

Detailed results are provided for the best-performing open-source and commercial models (Table 3). GPT-5 without guidelines achieved an *R*^2^ of 0.93 with *κ* = 0.700. When guidelines were provided, *R*^2^ decreased to 0.89 with *κ* = 0.640, reflecting only minor variation relative to the unguided condition. GPT-OSS-120B without guidelines reached an *R*^2^ of 0.92 with *κ* = 0.672. With guideline-based prompting, GPT-OSS-120B achieved the highest observed agreement, with an *R*^2^ of 0.93 and *κ* = 0.748. However, the difference between the 2 prompting strategies was statistically not significant (*P* = .68) for GPT-OSS-120b (cf. Appendix).

**Table 3.** Interclass agreement.

	Accuracy	*R* ^2^	Cohen’s *κ*
**Gpt-oss-120b without guidelines**	0.72	0.92	0.67
**Gpt-oss-120b with guidelines**	0.785	0.93	0.75
**Gpt-5 without guidelines**	0.745	0.93	0.70
**Gpt-5 with guidelines**	0.695	0.89	0.64

Accuracy, *R*^2^ values, and Cohen’s kappa for best-performing open-source and commercial LLM.

Abbreviation: LLM, large language model.

Taken together, both GPT-5 and GPT-OSS-120B demonstrated robust agreement with the human consensus across prompting strategies. The corresponding confusion matrices for both models are provided in Figure 2.

**Figure 2.**
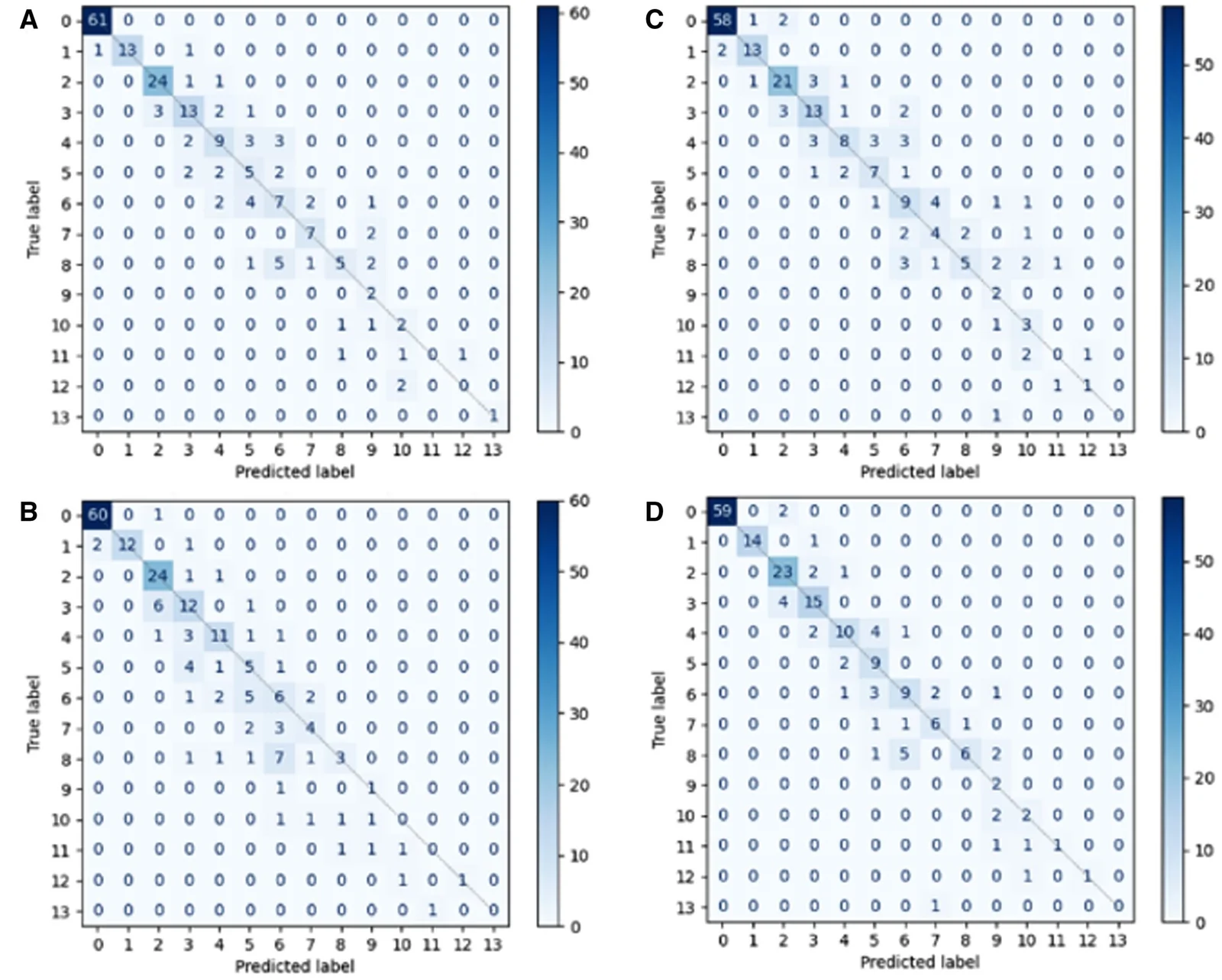
Confusion matrix for the best-performing commercial and open-source model. (A) GPT-5 without guidelines, (B) GPT-5 with guidelines, (C) GPT-oss 120b without guidelines, and (D) GPT-oss 120b with guidelines..

Agreement between LLM predictions and the human consensus was further analyzed by stratifying results according to ASA score (Table 4). For GPT-5 without guidelines, agreement decreased with increasing ASA score, showing perfect alignment in ASA I (accuracy = 0.98, *κ* = 0.9206, *R*^2^ = 0.823) and markedly lower agreement in ASA IV (accuracy = 0.46, *κ* = 0.3844, *R*^2^ = 0.750). For GPT-5 with guidelines, a similar decline was observed, with strong agreement in ASA I (accuracy = 0.98, *κ* = 0.9206, *R*^2^ = 0.823) and reduced agreement in ASA IV (accuracy = 0.34, *κ* = 0.2455, *R*^2^ = 0.735). GPT-OSS-120B achieved high agreement across all ASA categories, both with and without guidelines. Without guidelines, accuracies ranged from 1.000 in ASA I (*κ* = 1.000, *R*^2^ = 1.000) to 0.36 in ASA IV (*κ* = 0.2661, *R*^2^ = 0.647). With guidelines, performance was again strong, with accuracies of 0.98 (*κ* = 0.9206, *R*^2^ = 0.823) in ASA I and 0.56 (*κ* = 0.4977, *R*^2^ = 0.757) in ASA IV.

**Table 4.** Agreement between LLM predictions and human consensus across ASA scores.

	ASA	Accuracy	Cohen’s *κ*	*R* ^2^
**Gpt-oss-120b without guidelines**	1	1.00	1.00	1.00
	2	0.86	0.82	0.86
	3	0.66	0.62	0.91
	4	0.36	0.27	0.65
**Gpt-oss-120b with guidelines**	1	0.98	0.92	0.82
	2	0.92	0.90	0.92
	3	0.68	0.64	0.89
	4	0.56	0.50	0.76
**Gpt-5 without guidelines**	1	0.98	0.92	0.82
	2	0.92	0.90	0.95
	3	0.62	0.57	0.87
	4	0.46	0.38	0.75
**Gpt-5 with guidelines**	1	0.98	0.92	0.82
	2	0.86	0.82	0.87
	3	0.6	0.55	0.85
	4	0.34	0.25	0.73

Accuracy, Cohen’s *κ* and *R*^2^ are reported for GPT-5 (with and without guidelines) and GPT-OSS-120B (with and without guidelines).

Abbreviations: ASA, American Society of Anesthesiologists; LLM, large language model.

### Error analysis

Discrepancies between LLM-derived and human consensus CCI classifications were observed in 21.5%-30.5% of cases across all models.

For GPT-OSS-120B without guidelines, discrepancies occurred in 55 of 200 cases (27.5%). Following case-by-case review of these 55 discrepancies, 18.2% of disagreements reflected divergent interpretations of CCI criteria and ultimately aligned with the LLM output, 49.1% aligned with the human consensus, and in 32.7% no definitive CCI classification could be established. When guideline-based prompting was applied, GPT-OSS-120B showed 43 discrepancies (21.5%), with 25.6% of those aligning with the LLM interpretation, 51.2% with the human consensus, and 23.3% remaining indeterminate due to ambiguous diagnostic input.

For GPT-5 without guidelines, 51 discrepant cases (25.5%) were identified, of which 33.3% aligned with the LLM interpretation, 43.1% with the human consensus, and 23.5% remained inconclusive. In the GPT-5 with guidelines configuration, 61 discrepancies (30.5%) were found, with 16.4% aligning with the LLM interpretation, 60.7% with the human consensus, and 23.0% classified as indeterminate.

Across all models, discrepancies increased with higher ASA categories. Fewer than 3% of disagreements occurred in ASA I, while ASA III and ASA IV accounted for the majority of errors (29%-54% depending on model and configuration).

Based on the error classification framework by Khandekar et al.,^8^ most discrepancies were attributed to type A (knowledge-related) errors, comprising 41.7%-58.3% across models. Type B (parameter extraction) errors accounted for 12.0%-29.2%, while type C (computation) errors were rare or absent (0%-10%). Type D errors occurred in 22.2%-32.0% of discrepant cases and were exclusively observed in instances where no consistent CCI classification could be derived because the diagnosis list lacked sufficient clarity or completeness. A diagnosis list was judged insufficient when at least 1 documented condition could plausibly be mapped to CCI items carrying different weights and the list contained no information allowing a decision between them, so that more than 1 CCI value was defensible. Typical examples were renal insufficiency documented without stage or laboratory values, so that it could not be determined whether the criteria for moderate or severe renal disease (2 points) were met, and diabetes mellitus documented without information on treatment, such as insulin dependence, or on end-organ damage, which determines whether and with which weight the condition is counted. Examples of discrepant cases, including an extraction error by the LLM and an error by the human raters, are shown in Figures S1-S3 (Figure 3).

**Figure 3.**
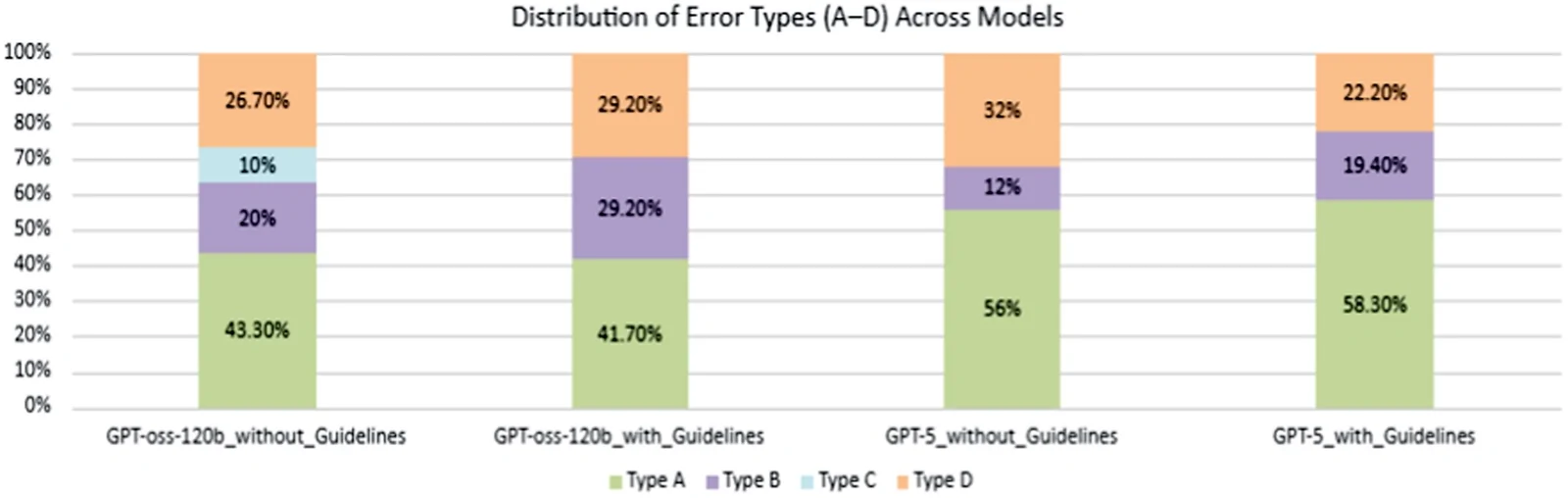
Distribution of error types across models. Type A errors represent knowledge-related mistakes, type B extraction errors, type C computation errors, and type D other or unspecified errors. Percentages indicate the relative frequency of each error type among all identified discrepancies.

## Discussion

Accurately identifying patient comorbidities is essential for clinical research, risk adjustment, and outcome prediction, yet standardized comorbidity scores such as the CCI are rarely available in real-world EHRs. Large language models offer a scalable alternative by extracting and structuring diagnostic information from unstructured clinical text. This study evaluated whether LLMs can compute the CCI directly from German-language diagnosis lists with reliability comparable to expert consensus and demonstrates that they can achieve accuracy and agreement approaching expert-level performance. Across 17 evaluated systems, both commercial and open-source models achieved substantial concordance with human consensus (accuracy up to 78.5%, *κ* up to 0.75). Human raters themselves showed excellent reproducibility (ICC = 0.97), confirming the robustness of the reference standard^9^^,^^10^ and supporting the use of LLMs as scalable, reliable tools for automated comorbidity assessment in German-language clinical text.

These results extend prior research, conducted predominantly in English and more recently in other languages including Spanish, German, and Turkish, showing that instruction-guided LLMs encode transferable clinical knowledge and can extract structured information from narrative texts.^3–5^ By applying models directly to German diagnosis lists, this study provides evidence that general-purpose LLMs can accurately interpret German free-text diagnosis lists without translation or ICD-coding intermediaries. Whether these results transfer to other languages, particularly lower-resource languages that are less represented in model training data, remains to be established. With GPT-OSS-120b, an open-source model achieved results comparable to the best-performing commercial models. Such open-source models can be hosted using local infrastructure within the hospital premises which allows for using such methods on real-world data without data privacy concerns.

Evidence on automated CCI derivation from clinical text in other languages remains limited and heterogeneous in task definition. In English, MedCalc-Bench includes the CCI among its calculators and evaluates LLMs on patient notes derived from published case reports,^8^ and Kara and Gunel evaluated publicly available LLMs on 5 clinical risk scores, including the CCI, calculated from synthetic clinical notes.^11^ For French, Petit-Jean et al. developed a hybrid rule-based and transformer pipeline that detects 16 Charlson conditions in clinical notes, rather than computing the score end-to-end with a generative model.^12^ For Italian, Di Maio et al. recently compared small and LLMs, including locally deployable models, with clinician-derived CCI and ASA scores in orthopedic surgical patients.^7^ Related work on comorbidity extraction with generative models has been reported for Spanish oncology reports^3^ and Turkish clinical records,^5^ and on German clinical documentation more broadly.^4^ Because datasets, reference standards, CCI versions, and agreement metrics differ across these studies, their results are not directly comparable with ours; a shared multilingual benchmark for comorbidity scoring would help to establish the current state of the art across languages.

### Interrater reliability

Human raters achieved near-perfect agreement when applying CCI rules by consensus (*κ* = 0.81-0.87, ICC = 0.97), meeting established interpretive thresholds for reliability and supporting use of expert consensus as a high-quality reference.^9^^,^^10^ These findings align with prior evaluations confirming the CCI’s strong clinimetric properties and reproducibility.^1^^,^^2^ Despite heterogeneous free-text input, trained clinicians could consistently interpret comorbidity categories, supporting the robustness of the expert consensus.

This demonstrates that manual CCI scoring can be performed with high reliability when standardized criteria and rater calibration are applied, even in unstructured diagnosis lists. Accordingly, the expert consensus offers a solid benchmark for evaluating LLM-derived classifications and underscores the potential of automating this otherwise time-consuming process.

### LLM-human consensus agreement

Agreement between LLM-derived and human consensus CCI scores was generally high (most *R*^2^ > 0.80). As in prior studies, large instruction-tuned systems exhibited strong medical knowledge and reasoning, with proprietary models showing the most stable concordance.^13–16^ Open-source models displayed wider variance—consistent with differences in scale, domain alignment, and instruction-following capabilities.^17^ Importantly, guideline-based prompting improved performance for most open models, narrowing gaps and enhancing reliability, which echoes controlled investigations of prompt design and zero-shot strategies in clinical natural language processing (NLP).^18–21^ For highly aligned proprietary models, incremental benefit from additional task guidance was smaller, suggesting that instruction-following schemas are already internalized.^20–22^

### Error analysis

Approximately one-quarter of records showed discrepancies between model- and human-derived CCI scores, a rate comparable to prior EHR information-extraction studies.^3^^,^^23^ In roughly one-third of these discrepant cases, the model correctly identified comorbidities that the human consensus missed or misclassified, suggesting complementary strengths between automated and manual approaches.

Most discrepancies were knowledge-related (type A), reflecting incomplete or inaccurate application of CCI rules—patterns consistent with known reasoning limitations of current LLMs.^6^^,^^13^^,^^16^ Extraction errors (type B) primarily resulted from nonstandard abbreviations, local terminology, or ambiguous phrasing in diagnosis lists.^4^^,^^5^ Computation errors (type C) were rare, confirming that models generally aggregated points accurately once the parameters were recognized. The relatively high proportion of type D errors reflects a limitation of both the input data and the evaluation process rather than a limitation of the models themselves. In our case, type D errors arose exclusively due to incomplete or inaccurate diagnosis lists.

It is plausible that LLMs may handle such imperfect or variably documented clinical inputs more consistently than human raters, as they apply learned decision patterns uniformly across cases rather than relying on case-specific and subjective heuristics. This systematic processing could help explain observations where model outputs align well—or occasionally more closely than expected—with reference annotations in less complete records. Similar patterns have been noted in recent evaluations showing that LLM-based extraction systems can maintain internally consistent outputs and stable performance across unstructured or heterogeneous clinical text sources.^24^

Error frequency increased with higher ASA categories, reflecting a dual mechanism of rising clinical complexity and deteriorating documentation quality. As ASA severity rose, diagnosis lists tended to contain more entries yet were often incomplete, inconsistently formatted, or poorly specified—conditions that hinder accurate CCI determination even for expert raters. This pattern aligns with prior evidence that documentation workflow and system design materially influence diagnostic accuracy and completeness—for example, agreement between coded diagnoses and charted notes nearly doubled when documentation processes were integrated rather than separate.^25^

Moreover, even structured “problem lists” in EHRs—while generally accurate for individual diagnoses—frequently omit or misrepresent additional conditions, thereby underestimating overall multimorbidity.^26^ Broader evaluations of digital health data quality similarly reveal pervasive issues of incompleteness, inconsistency, and implausible entries, all of which can substantially affect downstream research and analytic reliability.^27^

Taken together, these findings indicate that inadequate real-world documentation—especially in unstructured diagnosis lists—remains a major source of CCI-scoring error, often outweighing purely model-related deficits. Improving documentation quality through standardized templates, integrated workflows, and interoperable data-capture tools is therefore likely to yield substantial gains for both human and LLM-based comorbidity assessment.

### Strengths and limitations

Several methodological and contextual strengths underpin this study. A major strength lies in the robust human reference standard: 3 independent raters achieved near-perfect agreement (*κ* = 0.81-0.87; ICC = 0.97),^9^^,^^10^ providing a dependable benchmark for model comparison. The evaluation also included both commercial and open-source systems under identical prompting conditions, allowing transparent assessment of generalizability. Balanced stratification across ASA classes enabled analysis of performance under increasing clinical complexity, while the use of multiple concordance metrics (accuracy, *κ*, *R*^2^) aligns with best-practice recommendations for reliability research.^9^^,^^10^

Despite these strengths, several limitations should be acknowledged.

First, no explicit version of the CCI (eg, original or updated weighting) was specified. Because CCI versions differ in inclusion rules and point assignments,^1^^,^^2^ an explicit version might have improved consistency and interpretability for both raters and models, potentially enhancing LLM performance.

Second, the prompts and diagnosis lists used for LLM querying were not exhaustive. They provided only partial or generic guidance, which may have limited the models’ ability to fully map comorbidities to CCI categories—particularly for nuanced distinctions such as disease severity. This incomplete task framing likely contributed to residual knowledge-related errors, consistent with prior reports that prompt specificity strongly influences reasoning accuracy.^18–21^

Third, the study was conducted in a single language (German) at a single orthopedic center. German is a comparatively high-resource language in NLP, and our findings should not be generalized to other languages, particularly lower-resource ones, or to other specialties and documentation cultures without dedicated validation.

Fourth, the review of discrepant cases was performed by the same raters who established the reference standard, and it was not blinded to the source of each score. This may have biased the attribution of errors in favor of the human consensus; nevertheless, 16%-33% of discrepancies were resolved in favor of the LLM output.

None of the models were fine-tuned on German clinical corpora. Domain-specific pretraining, as demonstrated for biomedical transformers such as BioBERT,^17^ could further improve extraction accuracy in German-language EHRs.

Finally, continual model updates may influence reproducibility, emphasizing the need for transparent version documentation and longitudinal benchmarking.

Together, these factors contextualize the present results and outline opportunities for improvement in future LLM-based comorbidity research.

## Conclusion

In summary, LLMs can approximate expert-level CCI computation from unstructured German EHRs. Both open- and closed-source systems demonstrated substantial agreement with human consensus, and structured prompting further improved accuracy. While knowledge-related reasoning errors persist, most discrepancies stemmed from documentation gaps or incomplete task specification rather than inherent model failure. Addressing these factors—by specifying standardized CCI versions, refining prompts, and improving data completeness—could yield even greater reliability. Large language model-assisted comorbidity scoring thus represents a reproducible, scalable tool for accelerating clinical research and quality analytics in German-language health-care settings, with validation in other languages warranted.

## Supplementary Material

ooag212_Supplementary_Data

## Data Availability

The datasets generated and/or analyzed during the current study are not publicly available but are available from the corresponding author on reasonable request. The code is available from the corresponding author on reasonable request.
