# Supplementary material for: Harnessing large language models for reliable automatic Charlson Comorbidity Index scoring: a validation study
Source: JAMIA Open. 2026 Nov 2;9(5):ooag212. doi: 10.1093/jamiaopen/ooag212 (PMC13726996; doi:10.1093/jamiaopen/ooag212)
Supplement: Supplementary file 1 [file ooag212_supplementary_data.docx]

**Appendix**

| **Model** | **Company** |
| --- | --- |
| *Open-source* | |
| MedGemma 27B | Google DeepMind, London, UK |
| DeepSeek R1 70B | DeepSeek AI, Beijing, China |
| DeepSeek V2.5 | DeepSeek AI, Beijing, China |
| Llama 3.1 70B | Meta Platforms, Inc., Menlo Park, CA, USA |
| Llama 3.3 | Meta Platforms, Inc., Menlo Park, CA, USA |
| Llama 3.3 | Meta Platforms, Inc., Menlo Park, CA, USA |
| Llama 4 Maverick 262k | Meta Platforms, Inc., Menlo Park, CA, USA |
| Meditron 70B (Q5_1) | École Polytechnique Fédérale de Lausanne (EPFL), Lausanne, Switzerland |
| Qwen3 235B | Alibaba Cloud Intelligence, Hangzhou, China |
| Qwen3 30B | Alibaba Cloud Intelligence, Hangzhou, China |
| GPT-OSS | OpenAI, San Francisco, CA, USA |
| GPT-OSS 120B | OpenAI, San Francisco, CA, USA |
| *Commercial* | |
| GPT-4.1 mini | OpenAI, San Francisco, CA, USA |
| GPT-4o | OpenAI, San Francisco, CA, USA |
| GPT-5 | OpenAI, San Francisco, CA, USA |
| Claude Opus 4.1 (2025-08-05) | Anthropic PBC, San Francisco, CA, USA |
| Claude Sonnet 4 (2025-05-14) | Anthropic PBC, San Francisco, CA, USA |

*Table A1:* Comprehensive list of Large Language Models used in this study

*Box A1:* Prompt template and CCI guidelines

The prompt below was used for the configuration with guidelines and is reproduced verbatim, including the original spelling. For the configuration without guidelines, the same template was used, but the CCI criteria in the Guidelines section (from “Myocardial infarction” to “AIDS”) were removed; the age groups with their points and all other sections were retained. The placeholders @@@AGE_GROUP@@@ and @@@DIAG@@@ were replaced by the age group and the complete German diagnosis list of each patient. The CCI criteria were formulated by the human raters during the trial run (see Methods). An English translation of the German passages follows.

```
Basierend auf der folgenden Liste, was ist der geschätzte Charlson Comorbidity Index (CCI) für einen Patienten in der Altersklasse @@@AGE_GROUP@@@ Jahre und mit folgenden Diagnosen?

### Alter:
Altersklasse @@@AGE_GROUP@@@ Jahre

=======

### Diagnosen:
@@@DIAG@@@

=======

### Guidelines:
Verwende ausschliesslich die Altersgruppe und die nachfolgenden Regeln. Für jede Diagnose, entscheide ob die Diagnose zu einem der nachfolgenden Kriterien passt.

---
Alterklassen:
  < 50 Jahre (0)
  50 - 59 Jahre (+1)
  60 - 69 Jahre (+2)
  70 - 79 Jahre (+3)
  80 - 89 Jahre (+4)
  >= 90 Jahre (+5)
---
Myocardial infarction (+1)
  (History of definite or probable MI (EKG changes and/or enzyme changes))
---
Congestive heart failure (+1)
  (Exertional or paroxysmal nocturnal dyspnea and has responded to digitalis, diuretics, or afterload reducing agents, LVEF < 40%, diastolic dysfuntion, pulmonary congestion)
---
Peripheral vascular disease (+1)
  (Intermittent claudication or past bypass for chronic arterial insufficiency, history of gangrene or acute arterial insufficiency, or untreated thoracic or abdominal aneurysm (>=6 cm))
---
Cerebrovascular disease (+1)
  (except hemiplegia, History of a cerebrovascular accident with minor or no residua and transient ischemic attacks)
---
Dementia (+1)
  (Chronic cognitive deficit)
---
Chronic pulmonary disease (+1)
  (Chronic bronchitis, Emphysema, Chronic ostructive asthma, Bronchiectasis)
---
Connective tissue disease (+1)
  (Rheumatoid Arthritis (RA), Systemic Lupus Erythematosus (SLE), Systemic Sclerosis (Scleroderma), Polymyositis/Dermatomyositis, Sjogren's Syndrome, Mixed Connective Tissue Disease (MCTD), Ankylosing Spondylitis (AS) and other spondyloarthropathies)
---
Peptic Ulcer disease (+1)
  (Any history of treatment for ulcer disease or history of ulcer bleeding, only PPI without ulcer is not enough)
---
Mild liver disease (+1)
  (mild = chronic hepatitis (or cirrhosis without portal hypertension))
---
Liverdisease moderate to severe (+3)
  (Severe = cirrhosis and portal hypertension with variceal bleeding history, moderate = cirrhosis and portal hypertension but no variceal bleeding history)
---
Diabetes (without complications) (+1)
---
Diabetes with end organ damage (+2)
---
Hemiplegia (+2)
---
Moderate or severe renal disease (+2)
  (Severe = on dialysis, status post kidney transplant, uremia, moderate = creatinine >3 mg/dL (0.27 mmol/L))
---
Solid tumor (non metastatic) (+2)
---
Leukemia (+2)
---
Lymphoma, Multiple myeloma (+2)
---
Metastatic solid tumor (+6)
---
AIDS (+6)
---
=======

### Instruktion:
Wichtig: Antworte nur mit einer Erklärung und dem CCI als JSON ("Explanation" und "CCI") ohne weitere Informationen.
```

**English translation of the German passages**

*Opening question:* Based on the following list, what is the estimated Charlson Comorbidity Index (CCI) for a patient in the age group @@@AGE_GROUP@@@ years with the following diagnoses?

*### Alter / ### Diagnosen:* ### Age: age group @@@AGE_GROUP@@@ years; ### Diagnoses: @@@DIAG@@@

*### Guidelines:* Use only the age group and the following rules. For each diagnosis, decide whether it matches one of the following criteria.

*Alterklassen:* Age groups: <50 years (0); 50–59 years (+1); 60–69 years (+2); 70–79 years (+3); 80–89 years (+4); ≥90 years (+5). The comorbidity criteria were written in English in the original prompt and are reproduced unchanged above.

*### Instruktion:* Important: respond only with an explanation and the CCI as JSON ("Explanation" and "CCI") without any further information.

*Error Examples 1*

*Figure A1: Real-world clinical diagnoses documented for the patient*

Human Consensus:

- Myocardial infarction (+1)
- Chronic pulmonary disease (+1)
- Age (+3)
- Total points = 5

GPT-5_without-guidelines:

{"Explanation":"Berechnung nach Charlson-Index (inkl. Alter):\n- Alter 70–79 Jahre: +3\n- Chronische Lungenerkrankung (COPD): +1\n- Keine Punkte für: KHK ohne dokumentierten Myokardinfarkt oder Herzinsuffizienz, Vorhofflimmern, Hypertonie, leichte Klappenvitien/Aortendilatation, Arthrose/Wirbelsäulenerkrankungen/Fraktur, Schlafapnoe, zurückliegende COVID-Pneumonie, frühere Lungenembolie.\nSumme: 4","CCI":4}
Error: Diagnosis list incomplete. Stent placement could be due to myocardial infarction or it could also be an elective procedure.

*Error Examples 2*

*Figure A2: Real-world clinical diagnoses documented for the patient*
Human Consensus:

- Diabetes (without complications)
- Age (+2)
- Total points = 4

GPT-oss-120b_with-guidelines:

{\n "Explanation": "The patient is in the 60‑69 age group, which adds +2 points. None of the listed diagnoses (spinal conditions, incontinence, surgeries, hypertension, or unspecified diabetes) correspond to any of the Charlson comorbidity categories provided. Therefore, the total CCI is solely the age contribution.",\n "CCI": 2\n}

Error: The abbreviation DM was not recognized by LLM as diabetes mellitus.

*Error example 3*

*Figure A3: Real-world clinical diagnoses documented for the patient*

Human Consensus:

- Age (+4)
- Total points = 4

GPT-5_without-guidelines:

{"Explanation":"Alter 80–89 Jahre: +4. Zerebrovaskuläre Erkrankung: St.n. Amaurosis fugax (entspricht TIA) → +1. Keine weiteren anrechenbaren CCI-Diagnosen (keine KHK/MI, Herzinsuffizienz, COPD, Ulkus, Leber-/Niereninsuffizienz, Diabetes, Tumor/Metastasen, Demenz, PAVK, connective tissue disease, AIDS). Gesamt: 5.","CCI":5}

Error: Overlooking or misjudging a diagnosis by human raters
